# TAK-861, a potent, orally available orexin receptor 2-selective agonist, produces wakefulness in monkeys and improves narcolepsy-like phenotypes in mouse models

**DOI:** 10.1038/s41598-024-70594-1

**Published:** 2024-09-06

**Authors:** Kayo Mitsukawa, Michiko Terada, Ryuji Yamada, Taku Monjo, Tetsuaki Hiyoshi, Masanori Nakakariya, Yuichi Kajita, Tatsuya Ando, Tatsuki Koike, Haruhide Kimura

**Affiliations:** 1 Neuroscience Drug Discovery Unit, Takeda Pharmaceutical Company Limited, 26-1, Muraoka-Higashi 2-Chome, Fujisawa, Kanagawa 251-8555 Japan; 2 Drug Metabolism and Pharmacokinetics Research Laboratories, Research, Takeda Pharmaceutical Company Limited, 26-1, Muraoka-Higashi 2-Chome, Fujisawa, Kanagawa 251-8555 Japan

**Keywords:** Orexin, Orexin receptor 2 agonist, TAK-861, TAK-994, Danavorexton, Narcolepsy type 1, Drug discovery, Neuroscience

## Abstract

Narcolepsy type 1 (NT1) is associated with severe loss of orexin neurons and characterized by symptoms including excessive daytime sleepiness and cataplexy. Current medications indicated for NT1 often show limited efficacy, not addressing the full spectrum of symptoms, demonstrating a need for novel drugs. We discovered a parenteral orexin receptor 2 (OX2R) agonist, danavorexton, and an orally available OX2R agonist, TAK-994; both improving NT1 phenotypes in mouse models and individuals with NT1. However, danavorexton has limited oral availability and TAK-994 has a risk of off-target liver toxicity. To avoid off-target-based adverse events, a highly potent molecule with low effective dose is preferred. Here, we show that a novel OX2R-selective agonist, TAK-861 [*N*-{(2*S*,3*R*)-4,4-Difluoro-1-(2-hydroxy-2-methylpropanoyl)-2-[(2,3′,5′-trifluoro[1,1′-biphenyl]-3-yl)methyl]pyrrolidin-3-yl}ethanesulfonamide], activates OX2R with a half-maximal effective concentration of 2.5 nM and promotes wakefulness at 1 mg/kg in mice and monkeys, suggesting ~ tenfold higher potency and lower effective dosage than TAK-994. Similar to TAK-994, TAK-861 substantially ameliorates wakefulness fragmentation and cataplexy-like episodes in orexin/ataxin-3 and orexin-tTA;TetO DTA mice (NT1 mouse models). Compared with modafinil, TAK-861 induces highly correlated brain-wide neuronal activation in orexin-tTA;TetO DTA mice, suggesting efficient wake-promoting effects. Thus, TAK-861 has potential as an effective treatment for individuals with hypersomnia disorders including narcolepsy, potentially with a favorable safety profile.

## Introduction

Orexin pathways are involved in multiple biological functions including sleep/wakefulness, metabolism, stress control, reward processes, and autonomic functions^1–3^. Two endogenous orexin peptides, orexin-A (OX-A) and orexin-B (OX-B), produced by orexin neurons in the lateral hypothalamus^1,2^, activate two post-synaptic G protein-coupled receptors (GPCRs), orexin receptor 1 (OX1R) and orexin receptor 2 (OX2R)^2^.

Narcolepsy type 1 (NT1) is associated with severe loss of orexin neurons and is characterized by multiple symptoms including excessive daytime sleepiness (EDS) and cataplexy^4,5^. OX2R knockout (KO) mice and canines with null mutations in the OX2R gene, but not OX1R KO mice, exhibit apparent NT1-like phenotypes including fragmentation of wakefulness and cataplexy-like episodes^6–8^, suggesting a pivotal role of OX2R in the pathophysiology of NT1.

Currently available medications for NT1 include the wake-promoting drugs modafinil, armodafinil, solriamfetol, methylphenidate, and amphetamines, drugs that primarily target transporters of dopamine and/or norepinephrine for EDS, and adrenergic and serotonergic reuptake inhibitors for cataplexy. However, these agents result in only partial reductions in symptoms, e.g., modafinil is effective for EDS but ineffective against cataplexy, and clomipramine is effective for cataplexy but may cause sedation^9–12^. Sodium oxybate, which targets GABA-B receptors, and pitolisant, which targets histamine H3 receptors, can improve EDS and cataplexy; however, they have potential for respiratory depression and insufficient efficacy, respectively^13,14^.

We previously discovered a parenteral OX2R agonist, TAK-925 (danavorexton)^15^, and an orally available OX2R agonist, TAK-994^16^. Both danavorexton and TAK-994 showed potent agonistic activity for recombinant human OX2R, with half-maximal effective concentration (EC_50_) values of 5.5 and 19 nM, respectively, and increased wakefulness in wild-type (WT) mice and monkeys. Moreover, both drugs ameliorated wakefulness fragmentation and cataplexy-like episodes in orexin neuron-ablated mouse models of NT1^16–18^. Consistent with these data, danavorexton significantly increased wakefulness in sleep-deprived healthy individuals^19^, and both danavorexton and TAK-994 significantly improved sleepiness and cataplexy in individuals with NT1^20,21^. However, danavorexton has limited oral availability, and TAK-994 was shown to have risk of drug-induced liver injury, resulting in termination of the phase 2 clinical studies for TAK-994. This TAK-994-induced liver toxicity was considered to be an off-target effect^20^. To avoid off-target-related adverse events, discovery of a drug with higher potency and lower effective dose than TAK-994 is preferred^22^. Here, we report preclinical characteristics of a novel, highly potent, orally available OX2R-selective agonist, TAK-861 [*N*-{(2*S*,3*R*)-4,4-Difluoro-1-(2-hydroxy-2-methylpropanoyl)-2-[(2,3′,5′-trifluoro[1,1′-biphenyl]-3-yl)methyl]pyrrolidin-3-yl}ethanesulfonamide].

## Results

### TAK-861 selectively activates OX2R

**Fig. 1:**
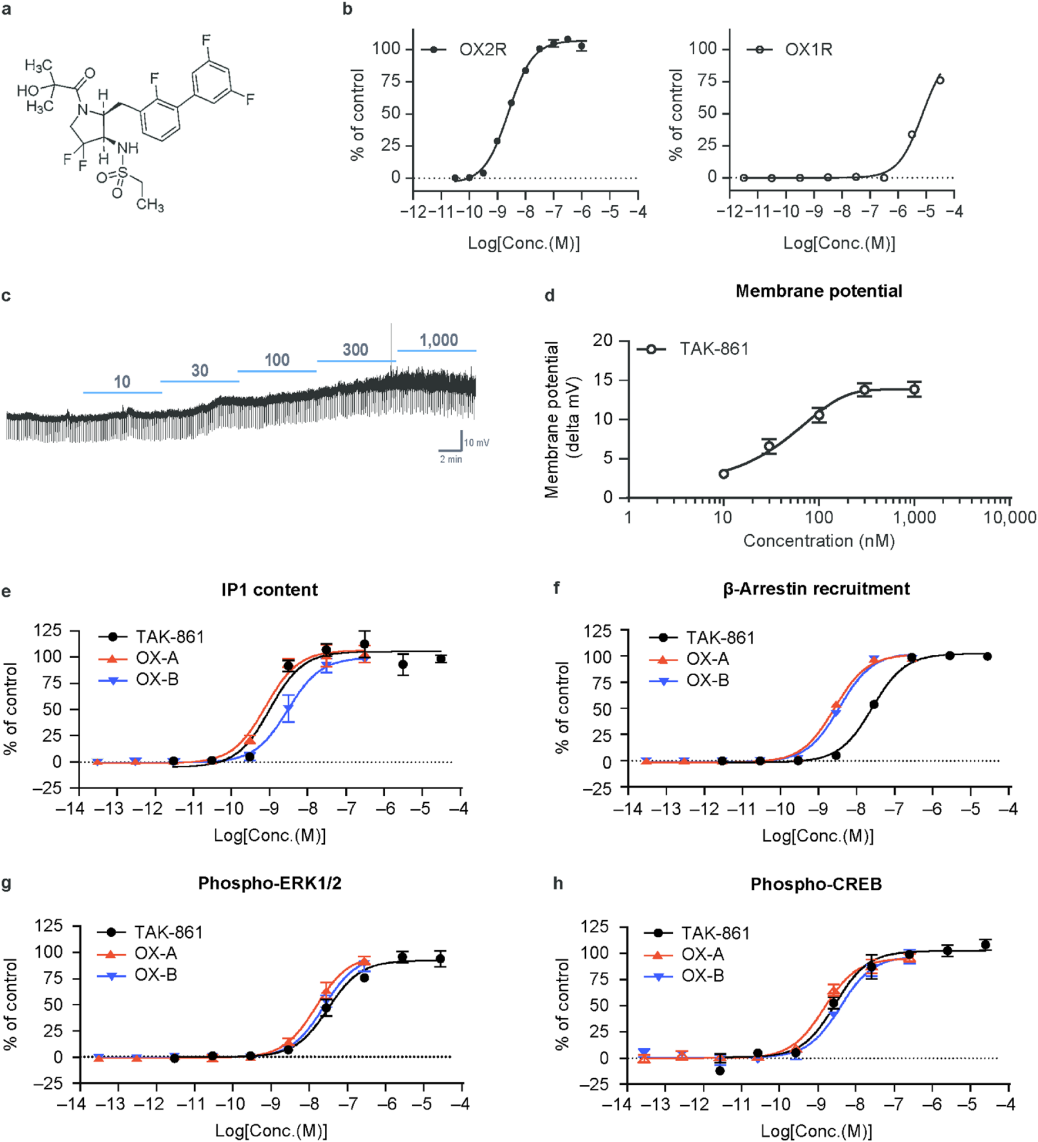
Activation of recombinant and physiological OX2R by TAK-861. (**a**) Chemical structure of TAK-861. (**b**) Effect of TAK-861 on calcium mobilization in hOX2R/CHO-K1 cells (left) and hOX1R/CHO-K1 cells (right). The responses to 100 nM OX-A represented the 100% response. Mean ± SD; n = 4. (**c**) Representative examples of membrane potential changes of histaminergic neurons in mouse tuberomammillary nucleus by TAK-861. (**d**) Dose–response changes in membrane potential by TAK-861. Each data point represents the average of six to eight recordings using brain slices obtained from seven mice. Mean ± standard error of the mean. (**e**) Effect of TAK-861 on intracellular accumulation of IP1 in hOX2R/CHO-EA cells. OX-A and OX-B were used as controls. The responses to 1 μM OX-A represented the 100% response. Mean ± SD; n = 4. (**f**) Effect of TAK-861 on β-arrestin recruitment in hOX2R/CHO-EA cells. OX-A and OX-B were used as controls. The responses to 1 μM OX-A represented the 100% response. Mean ± SD; n = 4. (**g**) Effect of TAK-861 on intracellular phosphorylation of ERK1/2 at Thr202/Tyr204. OX-A and OX-B were used as controls. The responses to 1 μM OX-A represented the 100% response. Mean ± SD; n = 4. (**h**) Effect of TAK-861 on intracellular phosphorylation of CREB at Ser133. OX-A and OX-B were used as controls. The responses to 1 μM OX-A represented the 100% response. Mean ± SD; n = 4. *CHO-K1* Chinese hamster ovary cells, *CREB* cAMP response element-binding protein, *ERK1/2* extracellular signal-regulated kinase 1/2, *hOX1R* human OX1R, *hOX2R* human OX2R, *IP1* inositol monophosphate, *OX-A* orexin-A, *OX-B* orexin-B, *OX1R* orexin receptor 1, *OX2R* orexin receptor 2, *SD* standard deviation.

The chemical structure of TAK-861 is shown in Fig. 1a. In calcium mobilization assays, TAK-861 potently activated recombinant OX2R (EC_50_ of 2.5 nM), whereas it weakly activated OX1R (EC_50_ of 7.5 × 10^3^ nM), indicative of its 3000-fold OX2R selectivity over OX1R (Fig. 1b and Table 1). Evaluation of the activity of TAK-861 on various enzymes, receptors, and ion channels (102 targets in total) in in vitro assays revealed that TAK-861 at 10 μM did not induce more than 50% inhibition or stimulation of these targets, except cannabinoid CB1 (58% inhibition) and progesterone receptor B (62% inhibition). With an EC_50_ of 2.5 nM to OX2R, TAK-861 is a highly potent OX2R-selective agonist with minimal off-target activity in vitro (Supplementary Tables S1 and S2).

**Table 1:** Comparison of in vitro potency and in vivo wake-promoting effects between TAK-861 and TAK-994. In vitro potency on recombinant hOX2R and the MED for wakefulness in WT mice and cynomolgus monkeys between TAK-861 and TAK-994^16,45^. *EC*_*50*_ half-maximal effective concentration, *hOX[1/2]R* human orexin receptor [1/2], *MED* minimum effective dose, *p.o.* orally, *WT* wild type.

	TAK-861	TAK-994
In vitro potency (hOX2R EC_50_)	2.5 nM	19 nM
Selectivity to hOX2R over hOX1R	× 3000	× 740
MEDs for wakefulness in WT animals		
Mice	1 mg/kg, p.o	10 mg/kg, p.o
Monkeys	1 mg/kg, p.o	10 mg/kg, p.o

Histaminergic neurons in the tuberomammillary nucleus (TMN) abundantly express OX2R but not OX1R^23,24^. TAK-861 induced dose-dependent depolarization of the membrane potential in histaminergic neurons in the mouse TMN with an EC_50_ of 31.7 nM (Fig. 1c,d), suggesting activation of physiological OX2R by TAK-861.

### TAK-861 activates signal transduction similar to those by orexin peptides

Inositol monophosphate (IP1) production, β-arrestin recruitment, and phosphorylation of extracellular signal-regulated kinase 1/2 (ERK1/2) and cAMP response element-binding protein (CREB) are involved in OX2R downstream signal transduction. In our previous studies, both danavorexton and TAK-994 activated these signals in OX2R-expressing cells^15,16^. OX-A, OX-B, and TAK-861 dose-dependently increased IP1 contents (EC_50_ of 0.84, 3.2, and 1.2 nM, respectively) (Fig. 1e), and increased β-arrestin recruitment (EC_50_ of 3.0, 4.0, and 30 nM) (Fig. 1f). OX-A, OX-B, and TAK-861 induced phosphorylation of ERK1/2 (EC_50_ of 17, 27, and 34 nM, respectively) (Fig. 1g) and phosphorylation of CREB (EC_50_ of 1.8, 4.7, and 3.6 nM) (Fig. 1h). Overall, these results suggest that TAK-861 activates OX2R downstream signals in a similar manner to orexin peptides.

### Oral administration of TAK-861 produces wake-promoting effects via OX2R activation in both mice and monkeys during the sleep phase

**Fig. 2:**
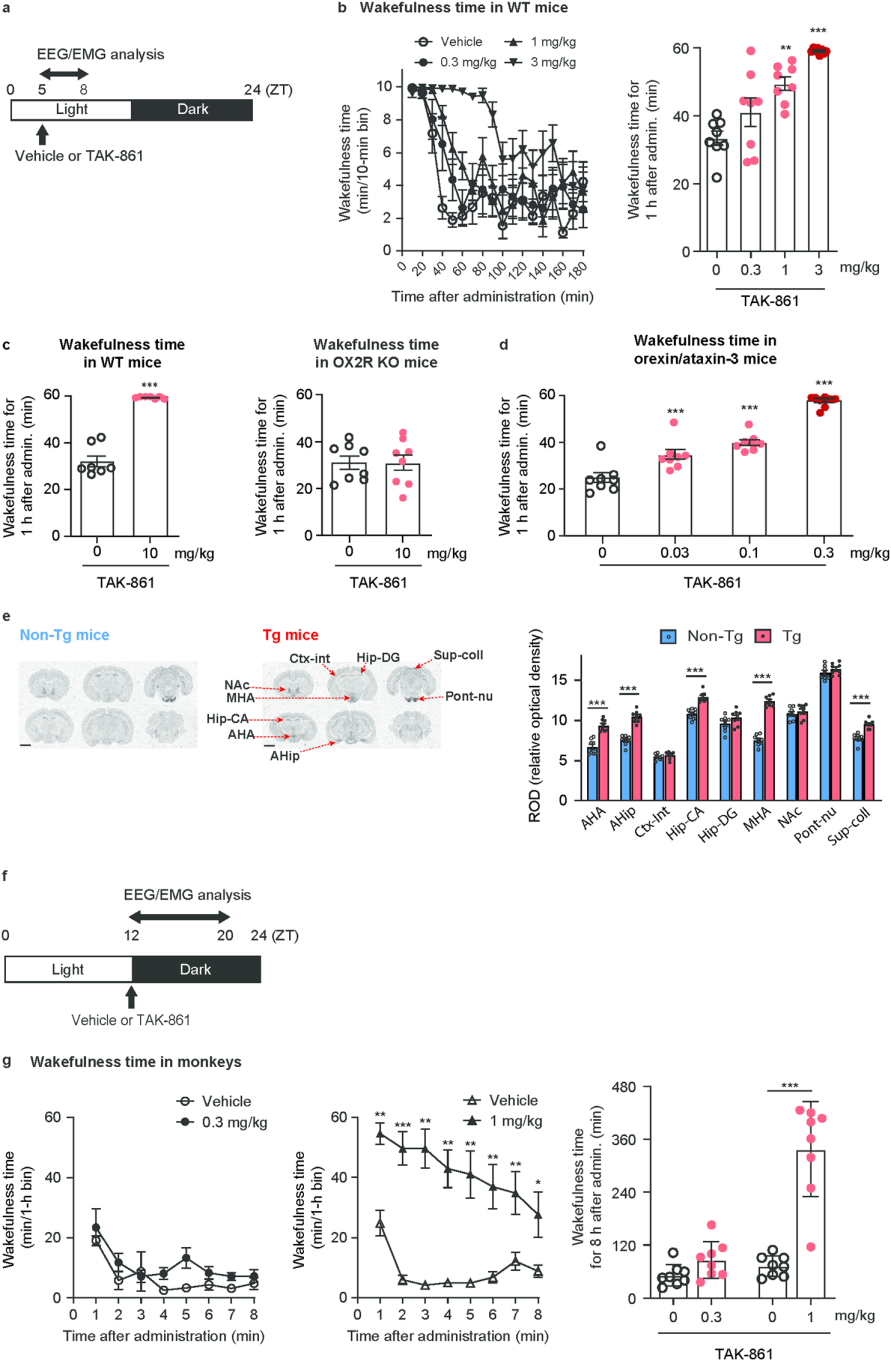
Wake-promoting effects of TAK-861 in WT mice, cynomolgus monkeys, and NT1 model mice during the sleep phase. (**a**) Time schedule of drug administration in mice during the sleep phase. TAK-861 or vehicle was administered orally to mice at ZT5, and then EEG/EMG and locomotor activity were recorded. (**b**) Effect of TAK-861 at 0.3, 1, and 3 mg/kg on wakefulness time in 10-min bins for 3 h (left) and total wakefulness time for 1 h (right) after administration in WT mice. Mean ± SEM; n = 8. **p < 0.01, ***p < 0.001, compared with the vehicle-treated mice, as determined by two-tailed Shirley-Williams test. (**c**) Effect of TAK-861 at 10 mg/kg on wakefulness time in WT mice (n = 7) and OX2R KO mice (n = 8). ***p < 0.001, compared with vehicle-treated mice, as determined by two-tailed paired *t*-test. (**d**) Effect of TAK-861 at 0.03, 0.1, and 0.3 mg/kg on wakefulness time in orexin/ataxin-3 mice. Mean ± SEM; n = 8. ***p < 0.001, compared with vehicle-treated mice, as determined by two-tailed Williams test. (**e**) Quantification of [^3^H]-EMPA binding in brain regions (arrows) in the autoradiograms of orexin/ataxin-3 mice and WT littermates (N = 6–8). The statistical analysis was performed between orexin/ataxin-3 mice and WT littermates using an analysis of variance (ANOVA) for repeated measures followed by post hoc two-tailed Student’s *t*-test with Bonferroni correction. ***p < 0.001. (**f**) Time schedule of drug administration in cynomolgus monkeys during the sleep phase. TAK-861 or vehicle was administered orally to mice at ZT12, and then EEG/EMG and locomotor activity were recorded. (**g**) Effects of TAK-861 on wakefulness time at 0.3 (left) and 1 mg/kg (middle) in 1-h bins for 8 h and total wakefulness time for 8 h (right) after administration in cynomolgus monkeys. Mean ± SEM; n = 8. Left and middle: *p < 0.05, **p < 0.01, ***p < 0.001, compared with the vehicle-treated mice, as determined by repeated measures analysis of variance followed by a post hoc two-tailed paired *t*-test, with multiplicity adjusted using the Holm method. Right: ***p < 0.001, compared with vehicle-treated cynomolgus monkeys in each group, as determined by paired *t*-test. *AHA* anterior hypothalamic, *AHip* amygdalohippocampal area, *Ctx-Int* internal layer of cortex, *EEG* electroencephalogram, *EMG* electromyogram, *Hip-CA* Cornu Ammonis (CA) of the hippocampus, *Hip-DG* dentate gyrus of the hippocampus, *KO* knockout, *MHA* medial hypothalamic area, *NAc* nucleus accumbens, *NT1* narcolepsy type 1, *Pont-nu* pontine nuclei, *SEM* standard error of the mean, *Sup-coll* superior colliculus, *WT* wild type, *ZT* zeitgeber time.

Wake-promoting effects of TAK-861 were assessed after oral administration in WT mice during the sleep phase (Fig. 2a). Duration of drug efficacy in mice is often different from that in humans because of the different pharmacokinetic profiles among species. Thus, the analysis time is determined according to the purpose of the study. In our previous studies, we analyzed the wake-promoting effects of danavorexton and TAK-994 during 1 h after administration in mice^16,21^. Time-dependent wake-promoting effects of TAK-861 in mice are shown in Fig. 2b. To compare the potency of TAK-861 with the potencies of TAK-925 and TAK-994, we assessed the wake-promoting effects of TAK-861 during 1 h after administration. TAK-861 at 1 and 3 mg/kg substantially increased total wakefulness time for 1 h after administration (Fig. 2b), accompanied by substantial decreases in total non-rapid eye movement (NREM) and rapid eye movement (REM) sleep times (Supplementary Fig. S1a,b). The minimum effective dose (MED) of TAK-861 for wakefulness in WT mice was 1 mg/kg (Fig. 2b and Table 1). To verify in vivo OX2R selectivity of TAK-861, the wake-promoting effect at 10 mg/kg was assessed in OX2R KO mice and their WT littermates (WT mice) during the sleep phase. In WT mice, TAK-861 substantially increased total wakefulness time (Fig. 2c), accompanied by a substantial decrease in total NREM sleep time (Supplementary Fig. S1c) and no change in total REM sleep time (Supplementary Fig. S1d). In contrast, TAK-861 did not affect total wakefulness time, total NREM sleep time, or total REM sleep time in OX2R KO mice (Fig. 2c and Supplementary Fig. S1e,f). Thus, TAK-861 would produce wakefulness via OX2R activation in mice.

In orexin/ataxin-3 mice, TAK-861 substantially increased total wakefulness time at 0.03, 0.1, and 0.3 mg/kg (Fig. 2d), accompanied by substantial decreases in total NREM sleep time (Supplementary Fig. S1g) and no change in total REM sleep time (Supplementary Fig. S1h). Thus, the MED of TAK-861 for wakefulness in orexin/ataxin-3 mice was ≤ 0.03 mg/kg, ≥ 30-fold lower than in WT littermates (1 mg/kg), as shown in Fig. 2b. Plasma pharmacokinetic profiles of TAK-861 (1 mg/kg) were comparable between WT and orexin/ataxin-3 mice (Supplementary Fig. S2). Accordingly, TAK-861 can produce wakefulness at a lower plasma concentration in orexin/ataxin-3 mice versus WT mice. This result is consistent with our previous observations for danavorexton and TAK-994^16,21^. One possible explanation for the higher sensitivity of orexin/ataxin-3 mice for OX2R agonists, compared with WT littermates, could be increased cell-surface expression of OX2R. By using in vitro autoradiography with an OX2R-selective radioligand, [^3^H] N-ethyl-2-[6-methoxy-pyridin-3-yl)-(toluene-2-sulfonyl)-amino]-N-pyridin-3-ylmethyl-acetamide (EMPA), we found significantly increased expression of OX2R protein in the anterior and medial hypothalamic area, amygdalohippocampal area, hippocampal CA region, and superior colliculus in orexin/ataxin-3 mice compared with WT littermates (Fig. 2e). Increased cell-surface expression of OX2R may contribute to higher sensitivity of orexin/ataxin-3 mice for wake-promoting effects of OX2R agonists, compared with WT mice.

Rodents have a polyphasic sleep/wake structure, whereas humans have monophasic sleep/wake patterns. Therefore, the wake-promoting effect of TAK-861 (0.3 or 1 mg/kg) was characterized in cynomolgus monkeys with monophasic sleep/wake patterns (Fig. 2f). Time-dependent wake-promoting effects of TAK-861 in monkeys are shown in Fig. 2g. In our previous studies, we analyzed the wake-promoting effects of danavorexton and TAK-994 during 8 h after administration in monkeys^19,45^. TAK-861 at 1 mg/kg, but not 0.3 mg/kg, considerably increased wakefulness time during 8 h after administration (Fig. 2g), suggesting that the MED of TAK-861 was 1 mg/kg for wake-promoting effects in cynomolgus monkeys (Table 1).

### TAK-861 ameliorates narcolepsy-like phenotypes in orexin/ataxin-3 mice

**Fig. 3:**
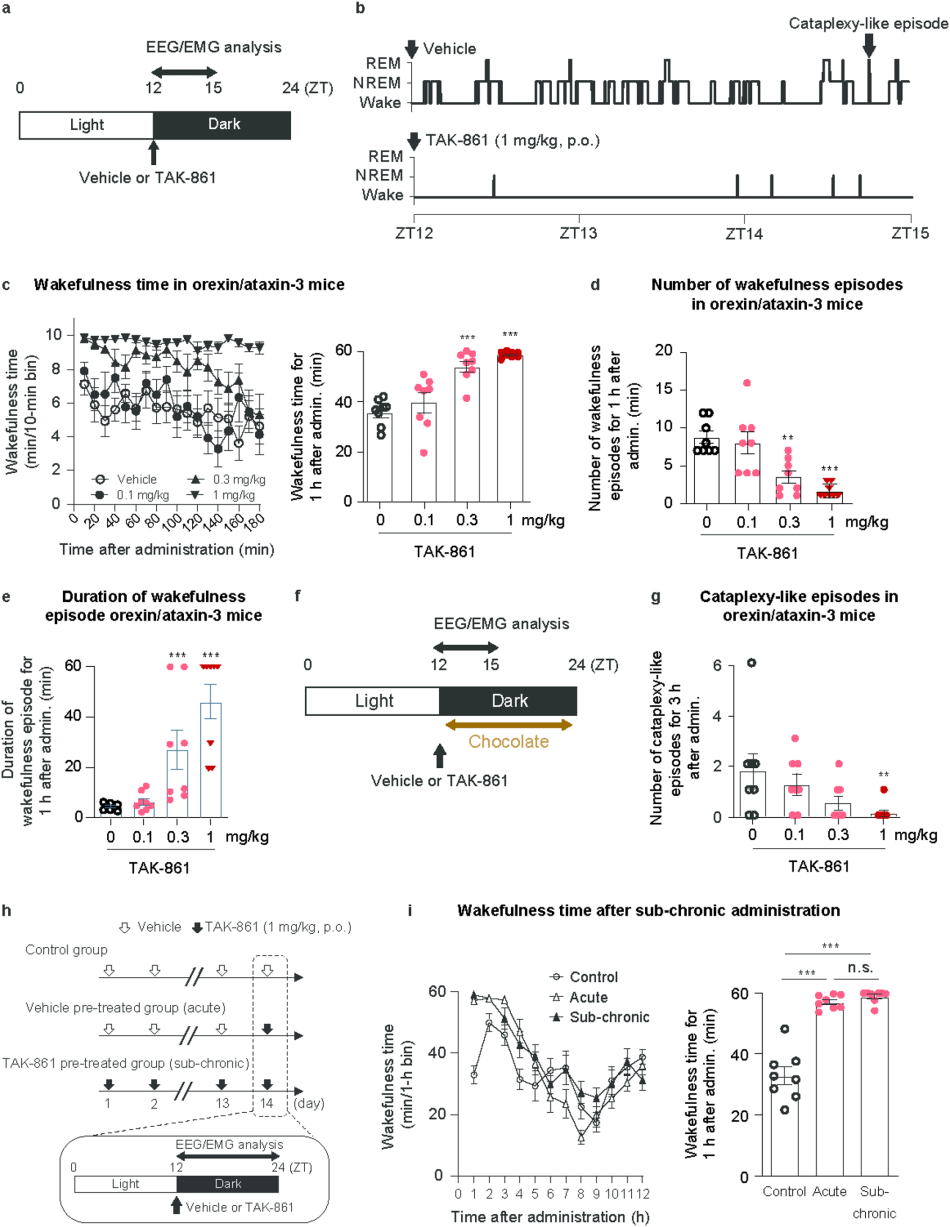
Effects of TAK-861 on narcolepsy-like symptoms in orexin/ataxin-3 mice during the active phase. (**a**) Time schedule of drug administration in orexin/ataxin-3 mice during the active phase for evaluation for sleep/wakefulness states. TAK-861 or vehicle was administered orally to mice at ZT12, and then EEG/EMG and locomotor activity were recorded. (**b**) Representative hypnogram for 3 h after TAK-861 (1 mg/kg) or vehicle administration in orexin/ataxin-3 mice. (**c**) Effect of TAK-861 (0.1, 0.3, and 1 mg/kg) on wakefulness time in 10-min bins for 3 h (left) and total wakefulness time for 1 h (right) after administration in orexin/ataxin-3 mice. Effect of TAK-861 (0.1, 0.3, and 1 mg/kg) on (**d**) number of wakefulness episode and (**e**) mean duration of wakefulness episode for 1 h after administration in orexin/ataxin-3 mice. Mean ± SEM; n = 8. **p < 0.01, ***p < 0.001, compared with vehicle-treated mice, as determined by two-tailed Shirley-Williams test. (**f**) Time schedule of drug administration in orexin/ataxin-3 mice during the active phase for evaluation of cataplexy-like episodes. After drug administration, chocolate was placed in the cage. The number of cataplexy-like episodes was determined for 3 h after administration. (**g**) Effect of TAK-861 (0.1, 0.3, and 1 mg/kg) on cataplexy-like episodes in orexin/ataxin-3 mice. Mean ± SEM; n = 8. **p < 0.01, compared with vehicle-treated mice, as determined by two-tailed Shirley-Williams test. (**h**) Time schedule of repeated drug administration in mice during the active phase. In the control or sub-chronic treatment group, vehicle or TAK-861 was orally administered to mice at ZT12 for 14 days, respectively. In the acute treatment group, vehicle was orally administered to mice at ZT12 for 13 days then TAK-861 was administrated on day 14. (**i**) Effects of acute and sub-chronic treatment of TAK-861 (1 mg/kg) on wakefulness time in 1-h bins for 12 h (left) and total wakefulness time for 1 h (right) after administration in orexin/ataxin-3 mice. Mean ± SEM; n = 8. ***p < 0.001, compared with vehicle-treated mice, as determined by Tukey’s multiple comparison test. *n.s.* not significant, *EEG* electroencephalogram, *EMG* electromyogram, *SEM* standard error of the mean, *ZT* zeitgeber time.

Orexin/ataxin-3 mice with selective loss of orexin neurons show narcolepsy-like symptoms such as wakefulness fragmentation and cataplexy-like episodes during the active phase^25^. Representative hypnograms for 3 h after oral administration of TAK-861 (1 mg/kg) or vehicle to orexin/ataxin-3 mice showed suppression of fragmented sleep by TAK-861 (Fig. 3a,b). In fact, TAK-861 time- and dose-dependently increased wakefulness time (Fig. 3c). Total wakefulness time was substantially increased (Fig. 3c), accompanied by considerable decreases in total NREM sleep and REM sleep times (Supplementary Fig. S3a,b) for 1 h after administration of TAK-861. TAK-861 also substantially ameliorated wakefulness fragmentation; both fewer number and longer mean duration of wakefulness episodes suggested less fragmented sleep (Fig. 3d,e).

The effect of TAK-861 on cataplexy-like episodes was examined for 3 h after administration in orexin/ataxin-3 mice in the presence of chocolate as emotional stimuli (Fig. 3f)^16,21^. As a result, TAK-861 (1 mg/kg) substantially suppressed cataplexy-like episodes (Fig. 3g). Thus, TAK-861 can ameliorate not only wakefulness fragmentation but also cataplexy-like episodes in orexin/ataxin-3 mice.

Chronic treatment of GPCR agonists often results in loss of efficacy due to receptor desensitization^26^. From our experience in drug discovery, the risk of GPCR desensitization becomes higher as the dose increases. Thus, we selected a high dose (1 mg/kg) to assess the potential risk of OX2R desensitization by TAK-861. The wake-promoting effect of TAK-861 after 14 days of sub-chronic dosing at 1 mg/kg at zeitgeber time (ZT) 12 was evaluated during the active phase in orexin/ataxin-3 mice (Fig. 3h). The plasma concentration of TAK-861 was comparable between acute and sub-chronic treatment groups (Supplementary Fig. S3c). To gain insight into wake/sleep structure after potent arousal by TAK-861, we measured electroencephalogram (EEG) for a longer period of 12 h after TAK-861 administration (Fig. 3i). Acute and sub-chronic treatment of TAK-861 substantially increased total wakefulness time (Fig. 3i) and decreased total NREM sleep and REM sleep time during 1 h after administration of TAK-861 (Supplementary Fig. S3d,e). There was no statistical difference in efficacy between the acute and sub-chronic treatment groups for 1 h after administration, suggesting that the wake-promoting effects of TAK-861 were maintained following sub-chronic dosing for up to 14 days in orexin/ataxin-3 mice. Although this study was performed in the absence of chocolate and not ideally designed to assess the effects of TAK-861 on cataplexy-like episodes after repeated administration, we analyzed the number of cataplexy-like episodes using the data from this study (Supplementary Fig. S3f.). Acute and sub-chronic administration of TAK-861 tended to decrease the number of cataplexy-like episodes to a comparable level, compared with vehicle administration. Thus, TAK-861 may have a low risk of OX2R desensitization. Notably, acute and sub-chronic treatment of TAK-861 showed no major difference in vigilance state during the subsequent sleep phase between ZT0 and ZT10; a substantial increase was only observed in total NREM sleep time after acute treatment of TAK-861 (Supplementary Fig. S3g). These findings suggest no sleep rebound after sub-chronic treatment with TAK-861.

### TAK-861 induces brain-wide neuronal activation in a highly correlated manner in orexin-tTA;TetO DTA mice

**Fig. 4:**
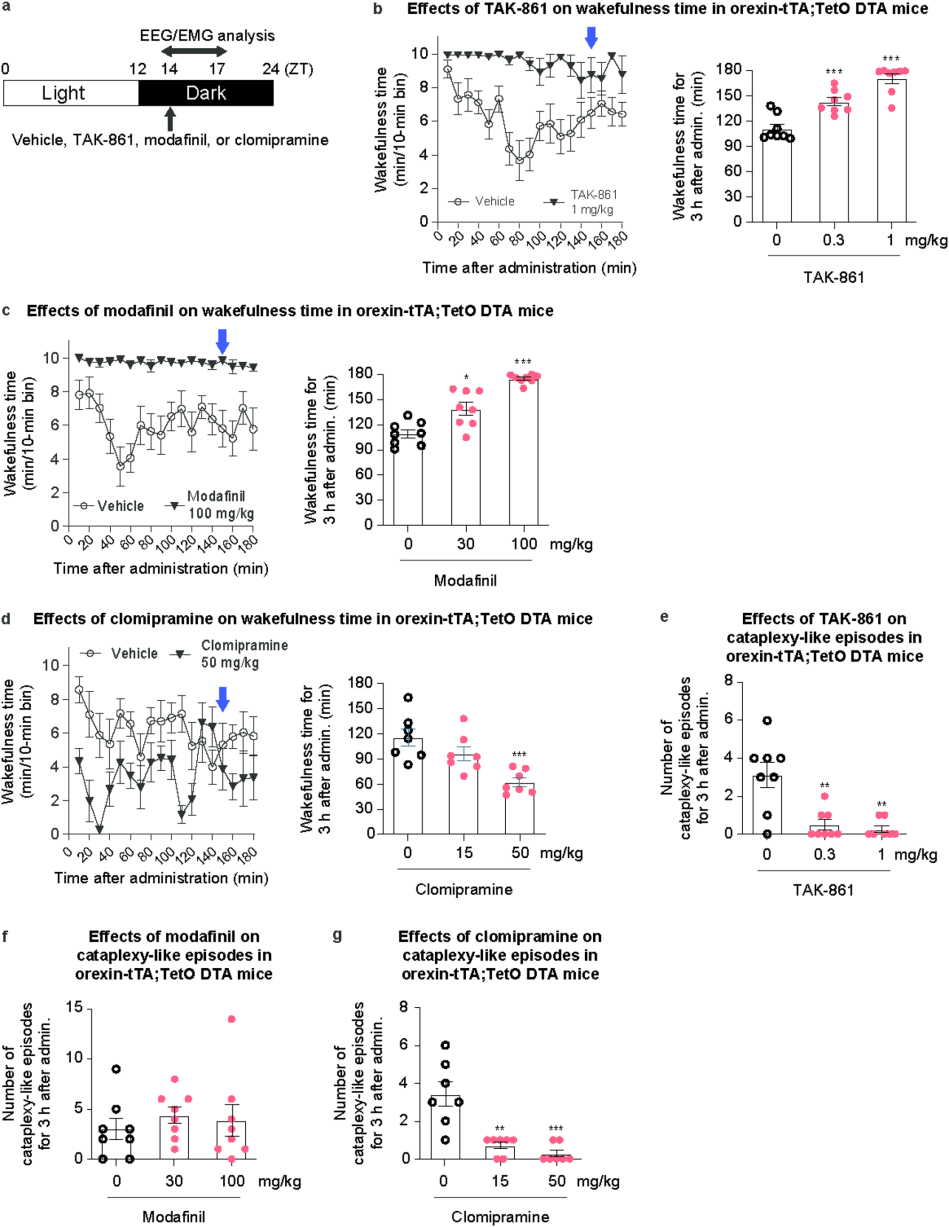
Effects of TAK-861, modafinil, and clomipramine on narcolepsy-like symptoms in orexin-tTA;TetO DTA mice during the active phase. (**a**) Time schedule of drug administration in orexin-tTA;TetO DTA mice during the active phase for evaluation for sleep/wakefulness states and cataplexy-like episodes. TAK-861, modafinil, clomipramine, or vehicle was administered orally to mice at ZT14, and then EEG/EMG and locomotor activity were recorded. Effects on wakefulness time of (**b**) TAK-861 (0.3 and 1 mg/kg), (**c**) modafinil (30 and 100 mg/kg), and (**d**) clomipramine (15 and 50 mg/kg) for 3 h after administration in orexin-tTA;TetO DTA mice. Blue arrows indicate the sampling time for the c-Fos mapping study in Fig. 5. Effects on cataplexy-like episodes of (**e**) TAK-861 (0.3 and 1 mg/kg), (**f**) modafinil (30 and 100 mg/kg), and (**g**) clomipramine (15 and 50 mg/kg) for 3 h after administration in orexin-tTA;TetO DTA mice. Mean ± standard error of the mean; n = 7–8. *p < 0.05, **p < 0.01, ***p < 0.001, compared with vehicle-treated mice, as determined by two-tailed Williams/Shirley-Williams test. *DTA* diphtheria toxin A, *EEG* electroencephalogram, *EMG* electromyogram.

OX2R-selective agonists showed substantial wake-promoting effects in NT1 model mice and individuals with NT1^17,21^. To understand the mechanisms of action underlying the potent efficacy of OX2R-selective agonists, we assessed brain-wide neuronal activation induced by TAK-861, modafinil, or clomipramine by whole-brain c-Fos immunohistochemistry in NT1 model mice. For this purpose, we worked to minimize neural activation through study conditions, such as drug administration procedure, lights-on/off, and chocolate. As orexin-tTA;TetO diphtheria toxin A (DTA) mice show more severe cataplexy-like episodes than orexin/ataxin-3 mice, cataplexy can be assessed in the absence of chocolate in orexin-tTA;TetO DTA mice. TAK-861 or vehicle was orally administered to orexin-tTA;TetO DTA mice at ZT14 (2 h after lights-off) because the effects of lights-off on c-Fos protein expression have been reported to disappear within 2 h^27^. To select the doses of drugs for whole-brain c-Fos immunohistochemistry, their effects on wakefulness and cataplexy-like episodes in the absence of chocolate were evaluated in orexin-tTA;TetO DTA mice (Fig. 4a). Both TAK-861 (0.3 and 1 mg/kg) and modafinil (30 and 100 mg/kg) substantially increased total wakefulness time (Fig. 4b,c) and ameliorated wakefulness fragmentation evidenced by fewer number (Supplementary Fig. S4a,c) and longer mean duration (Supplementary Fig. S4b,d) of wakefulness episodes during 3 h after administration. In contrast, clomipramine (50 mg/kg) considerably decreased total wakefulness time (Fig. 4d), accompanied by a considerable decrease in the number of wakefulness episodes without any change in their mean duration during 3 h after administration (Supplementary Fig. S4e,f). TAK-861 (0.3 and 1 mg/kg) and clomipramine (15 and 50 mg/kg), but not modafinil, substantially suppressed cataplexy-like episodes during 3 h after administration (Fig. 4e–g). To compare c-Fos expression induced by drugs with different mechanisms of action, it would be important to compare under conditions with equivalent efficacy. The wake-promoting effects of TAK-861 at 1 mg/kg and modafinil at 100 mg/kg were comparable (wakefulness time for 3 h after administration [mean ± standard error of the mean]: 170.26 ± 5.79 and 174.83 ± 1.88 min, respectively). In the c-Fos expression analysis, brains were fixed at 2.5 h after drug administration. The analysis of wakefulness time in 10-min bins for 3 h showed the comparable efficacy of TAK-861 (1 mg/kg) and modafinil (100 mg/kg) in wakefulness at 2.5 h after administration (sampling time) (Fig. 4b,c). Suppression of cataplexy-like episodes by TAK-861 at 1 mg/kg and clomipramine at 50 mg/kg was comparable (number of cataplexy-like episodes: 0.25 ± 0.16 and 0.29 ± 0.18, respectively). Based on these results, 1, 100, and 50 mg/kg of TAK-861, modafinil, and clomipramine, respectively, were used for whole-brain c-Fos expression analyses in orexin-tTA;TetO DTA mice.

**Fig. 5:**
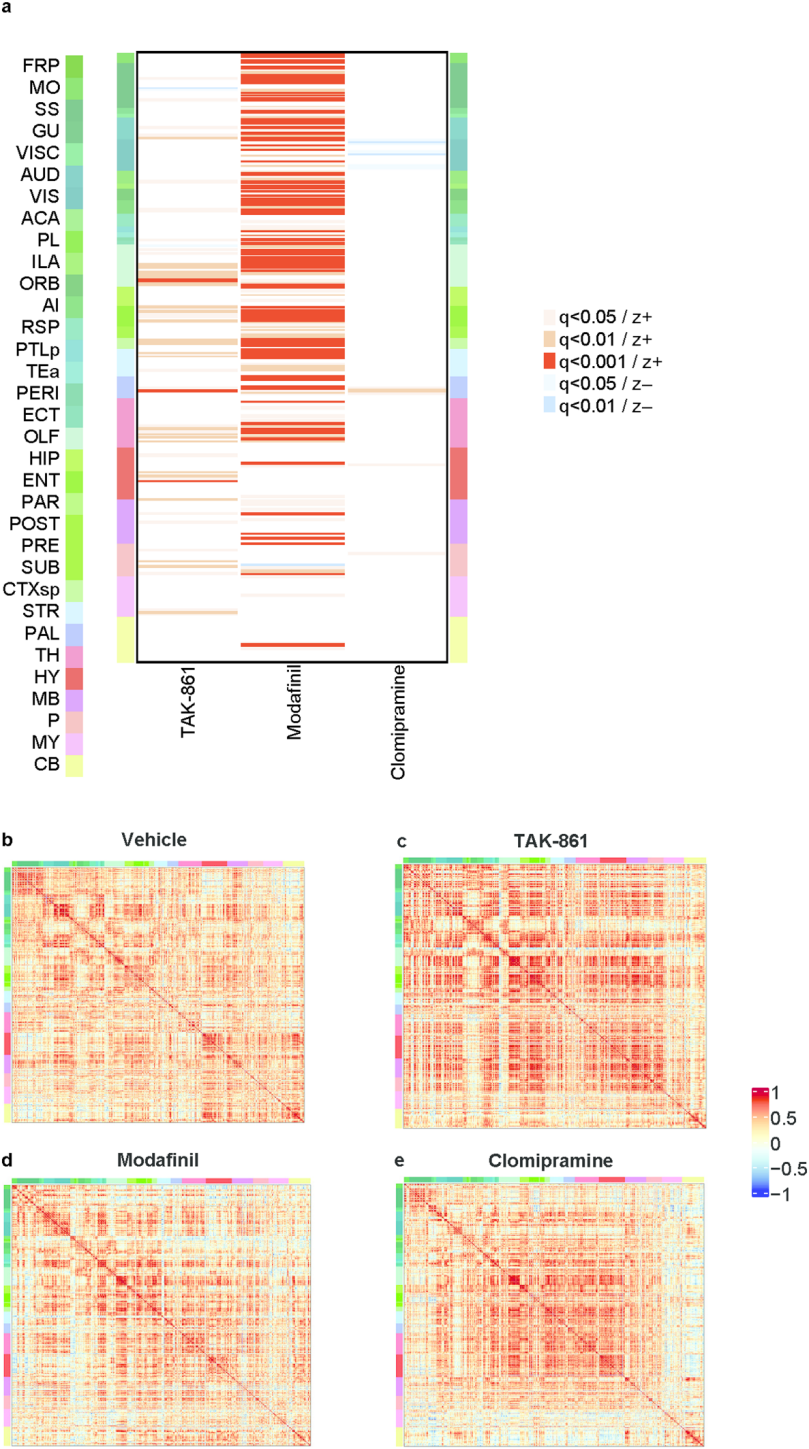
Brain-wide neuronal activity induced by TAK-861, modafinil, and clomipramine in orexin-tTA;TetO DTA mice during the active phase. (**a**) The heatmap shows significant changes (q < 0.05) of the c-Fos expression with the direction of z-score comparing with vehicle in the 747 regions of interest. The color bar shows the 33 brain categories and corresponding colors. The four pairwise correlation heatmaps were generated using Pearson’s correlation coefficients (− 1 to 1) from inter-regional c-Fos expression data across subjects for each group; (**b**) vehicle, (**c**) TAK-861, (**d**) modafinil, and (**e**) clomipramine. *ACA* anterior cingulate area, *AI* agranular insular area, *AUD* auditory areas, *CB* cerebellum, *CTXsp* cortical subplate, *DTA* diphtheria toxin A, *ECT* ectorhinal area, *ENT* entorhinal area, *FRP* frontal pole cerebral cortex, *GU* gustatory areas, *HIP* hippocampal region, *HY* hypothalamus, *ILA* infralimbic area, *MB* midbrain, *MO* somatomotor areas, *MY* medulla, *OLF* olfactory areas, *ORB* orbital area, *P* pons, *PAL* pallidum, *PAR* para-subiculum, *PERI* perirhinal area, *PL* prelimbic area, *POST* post-subiculum, *PRE* pre-subiculum, *PTLp* posterior parietal association areas, *RSP* retrosplenial area, *SS* somatosensory areas, *STR* striatum, *SUB* subiculum, *TEa* temporal association areas, *TH* thalamus, *VISC* visceral area, *VIS* visual areas.

TAK-861 (1 mg/kg) substantially increased the number of c-Fos-positive cells at various brain regions such as the cortical amygdala and TMN (Fig. 5a and Supplementary Table S3). Modafinil (100 mg/kg) more robustly increased the number of c-Fos-positive cells compared with TAK-861. Considering that 1 mg/kg TAK-861 and 100 mg/kg modafinil comparably increased wakefulness time in orexin-tTA;TetO DTA mice, TAK-861 can more efficiently produce wake-promoting effects than modafinil (i.e., potent wakefulness by low levels of neuronal activation). Clomipramine (50 mg/kg) substantially increased the number of c-Fos-positive cells only at bed nuclei of the stria terminalis, parabrachial nucleus, and parastrial nucleus. There were no brain regions where both TAK-861 and clomipramine, but not modafinil, induced neuronal activation, suggesting distinct mechanisms for suppression of cataplexy-like phenotypes between TAK-861 and clomipramine.

To further understand the efficient wake-promoting effects of TAK-861 in orexin-tTA;TetO DTA mice, pairwise correlation matrices were generated using Pearson’s correlation coefficients from inter-regional c-Fos expression data across mouse brain samples. When two regions (regions A and B) have similar patterns of c-Fos expression, with a correlation coefficient close to one, the relation between the regions is defined as having high correlation. In contrast, when two regions (regions B and C) have different patterns of c-Fos expression, with a correlation coefficient close to zero, the relation between the regions is defined as having low correlation (Supplementary Fig. S5). In the vehicle-treated group, a substantial correlation was observed from 966 region of interest (ROI) pairs. Under these conditions, TAK-861 induced an approximate tenfold increase, with substantial correlations from 11,019 ROI pairs (Fig. 5b,c). In contrast, modafinil and clomipramine did not increase the number of substantial correlations (778 and 827 ROI pairs, respectively); both drugs partly changed the correlated regions (Fig. 5d,e). Compared with modafinil, TAK-861 may induce neuronal activity with higher correlation across multiple brain regions to efficiently induce wake-promoting effects. After administration of clomipramine, correlation was observed in the limited brain regions, suggesting biased activation of the neural network. These findings suggest that TAK-861 induces brain-wide neuronal activation in a highly correlated manner to efficiently exert therapeutic effects in NT1 model mice.

## Discussion

Danavorexton, a parenteral OX2R-selective agonist, potently suppressed fragmentation of sleep and cataplexy-like episodes in NT1 model mice, and robustly increased sleep latency in the Maintenance of Wakefulness Test, up to the ceiling effect of 40 min, in individuals with NT1^17,21^. TAK-994, the first orally available OX2R-selective agonist, also suppressed fragmentation of sleep and cataplexy-like episodes in NT1 model mice, and improved measures of wakefulness and cataplexy in a phase 2 trial of individuals with NT1^20^. Therefore, OX2R agonists with similar properties to those of danavorexton and TAK-994 have the potential to produce potent efficacy in individuals with NT1. Because danavorexton has limited applicability for NT1 due to its parenteral administration and TAK-994 has a risk of off-target liver toxicity^20^, we aimed to identify a novel orally available OX2R agonist with similar pharmacological properties to those of danavorexton and TAK-994 but with an improved safety profile. The current hypothesis is that TAK-994–associated liver injury is caused by reactive metabolites and is likely an off-target effect of OX2R activation^20^. To avoid off-target-based adverse events, a highly potent molecule with low effective dose is preferred.

We found that TAK-861 (EC_50_ 2.5 nM; 3000-fold OX2R selectivity over OX1R) has approximately tenfold higher OX2R agonistic activity and fourfold higher OX2R selectivity than TAK-994 (EC_50_ 19 nM; 740-fold OX2R selectivity over OX1R) and activated similar signal transduction pathways to danavorexton and TAK-994^15,16^. TAK-861 produced considerable wake-promoting effects in WT mice and cynomolgus monkeys during the sleep phase, demonstrating wake-promoting effects in nocturnal mice and diurnal non-human primates at the physiological orexin level. Importantly, MEDs for wake-promoting effects of TAK-861 were ten times lower than MEDs of TAK-994 across species (Table 1). In two NT1 mouse models, orexin/ataxin-3 mice and orexin-tTA;TetO DTA mice, TAK-861 ameliorated wakefulness fragmentation and cataplexy-like episodes, consistent with observations for danavorexton and TAK-994^16,21^. Therefore, based on its much lower effective dose, the orally available OX2R-selective agonist TAK-861 has an improved off-target safety profile versus TAK-994. Based on these findings, TAK-861 could be expected to produce potent efficacy with a favorable safety profile in individuals with NT1.

To understand the mechanisms underlying the potent efficacy of OX2R agonists, brain-wide neuronal activity induced by TAK-861, modafinil, or clomipramine was assessed in orexin-tTA;TetO DTA mice. Although the precise mechanism of action of modafinil is unclear, both dopaminergic and noradrenergic systems are implicated in its action^28^. Clomipramine is a tricyclic antidepressant, which suppresses cataplexy by increasing the amount of serotonin and norepinephrine by inhibition of monoamine reuptake transporters^29^. In contrast to both modafinil and clomipramine, TAK-861 induced brain-wide neuronal activation in a highly correlated manner. This efficient neuronal activation could be associated with a low risk of reduced efficacy after chronic treatment and may be crucial for controlling a wide range of biological functions.

Orexin neurons, located in the lateral hypothalamus, receive inputs from various physiological stimuli, including the biological clock, sleep substances, and stressors^30^. They exert effects on multiple biological functions including wakefulness, locomotion, stress responses, attention, motivation, reward system, metabolism, pain, and autonomic function, likely owing to their wide projection to various brain regions, except the cerebellum^31,32^. Cross-talk between these biological responses may result in the coordination of brain-wide neuronal activities to synchronize multiple biological functions to achieve high-level appropriate behaviors. For example, an animal’s threat response would include increased wakefulness, locomotor activity, and attention, together with raised respiratory, cardiovascular, and metabolic responses, to improve their chance of survival. The hypothesis of OX2R as a hub for multiple behaviors and associated physiological responses is consistent with a wide distribution of OX2R protein in the brain^33^, although further elucidation of biological functions of OX2R is warranted. Together with an evaluation of functional recovery, in a future study, it would be interesting to investigate if the TAK-861-induced c-Fos expression pattern in NT1 model mice is similar to the pattern observed in WT mice.

Wake-promoting effects of orexin are quite impressive, and it should be considered that OX2R agonists would strongly and specifically activate brain regions associated with wakefulness, such as TMN, dorsal raphe (DR), and locus coeruleus (LC)^34^. However, orexin has multiple biological functions^1–3,35^. In our previous study, we showed the widespread distribution of OX2R protein in the rodent brain, except the cerebellum^33^, with high expression in several regions such as hippocampal dentate gyrus, cortical amygdala, nucleus accumbens (shell), and TMN, and moderate expression in various brain regions including DR and LC. Thus, an OX2R agonist could induce neuronal activation in the broader range of brain regions, not only specific to wakefulness-related regions related to the wakefulness^15^.

In summary, we found that TAK-861 was a highly potent and orally available OX2R-selective agonist in preclinical studies. Based on these findings, TAK-861 has the potential to provide potent efficacy in individuals with hypersomnia disorders including NT1, with a low risk of off-target adverse events. The clinical development of TAK-861 is currently underway (ClinicalTrials.gov Identifiers: NCT05687903, NCT05687916, NCT05816382).

## Methods

### Animals

Male C57BL/6J mice obtained from CLEA Japan Inc. (Tokyo, Japan) were used for slice electrophysiology studies at 3 weeks old according to the protocol (Protocol Number: AU-00030096) approved by the Institutional Animal Care and Use Committee (IACUC) of Shonan Health Innovation Park. Male OX2R KO mice were generated in our laboratory^15^ and used for EEG/electromyogram (EMG) studies at 18 weeks old according to the protocol (Protocol Number: AU-00030101) approved by the IACUC of Shonan Health Innovation Park. Orexin/ataxin-3 mice and orexin-tTA mice were obtained from University of Tsukuba^25^ and Nagoya University^36^, respectively. Male orexin/ataxin-3 mice and their WT littermates were bred in our laboratory and used for EEG/EMG studies at 22–29 weeks old according to the protocol (Protocol Number: AU-00020915) approved by the IACUC of Shonan Health Innovation Park. Orexin-tTA mice were crossed with TetO DTA mice (B6.Cg-Tg(tetO-DTA)1Gfi/J, The Jackson Laboratory, Bar Harbor, ME, USA) to generate orexin-tTA;TetO DTA mice^36^. Male orexin-tTA;TetO DTA mice were fed doxycycline (DOX)-containing chow (5TP7, Japan SLC, Inc., Shizuoka, Japan) until 6 weeks old, followed by being bred using DOX-free chow (CE-2, CLEA Japan Inc.) to induce loss of orexin neurons, and then used for EEG/EMG and c-Fos immunohistochemistry studies at 31–37 weeks old according to the protocol (Protocol Number: AU-00030665) approved by the IACUC of Shonan Health Innovation Park. All mice were housed under laboratory conditions (12-h light/dark cycles), with food and water available ad libitum.

Euthanasia was tolerated under the conditions if the following criteria were observed: more than 20% reduction of body weight, detachment of EEG/EMG electrodes, and/or obvious abnormalities such as seizure. At the end of the study, all mice were sacrificed by carbon dioxide exposure.

Male cynomolgus monkeys purchased from Hamri Co., Limited (Ibaraki, Japan) were housed under laboratory conditions (12-h light/dark cycles), with once-daily food and ad libitum water. They were used for EEG/EMG studies at 3 years old according to the protocol (Protocol Number: AU-00020938) approved by the IACUC of Shonan Health Innovation Park.

General procedures for animal care and housing were in accordance with the guidelines of the IACUC of Shonan Health Innovation Park, which is accredited by the Association and Accreditation of Laboratory Animal Care International. OX2R autoradiography analysis were conducted at Aptuit (Verona) Srl under approval of the internal Aptuit Committee on Animal Research and Ethics, with authorization issued by the Italian Ministry of Health (Italian Ministry of Health Authorization Project). All animal experiments were performed in accordance with ARRIVE guidelines.

### Chemicals

TAK-861 was synthesized by Takeda Pharmaceutical Company Limited. Modafinil was synthesized by LKT Laboratories, Inc. (St. Paul, MN, USA). Clomipramine and orexin peptides were purchased from Sigma-Aldrich (St. Louis, MO, USA) and Peptide Institute Inc. (Osaka, Japan), respectively. [^3^H]-EMPA (Specific Activity 79 Ci/mmol) was purchased from Novandi Chemistry AB (Södertälje, Sweden). JNJ-10397049 was synthesized in Aptuit Srl (Verona, Italy). 2-methylbutane was purchased from Fujifilm Wako Pure Chemical Co (Osaka, Japan). For in vitro studies, all drugs were dissolved in dimethyl sulfoxide (DMSO) (Fujifilm Wako Pure Chemical Co.) then diluted in each experimental solution. For in vivo studies, drugs were suspended in 0.5% (w/v) methylcellulose in distilled water (Fujifilm Wako Pure Chemical Co.) and were orally administered to mice at a volume of 10 mL/kg of body weight and to monkeys at a volume of 5 mL/kg of body weight.

### OX2R downstream signaling assay

Chinese hamster ovary-K1 (CHO-K1) cells (CCL-61, ATCC, Manassas, VA, USA) stably expressing human OX1R or OX2R (hOX1R/CHO-K1 cells or hOX2R/CHO-K1 cells) were used for calcium mobilization assay^15,16^. The responses to 0.5% DMSO in the absence and presence of 100 nM OX-A were used as 0% and 100% responses, respectively.

IP1 assay, β-arrestin recruitment assay, and evaluations of phosphorylation of ERK1/2 and CREB were conducted using a receptor cell line (hOX2R/CHO-EA) as described previously^15,16^. Intracellular accumulation of IP1 was measured using IP-One-Gq kit (Cisbio Bioassays, Bedford, MA, USA). The hOX2R/CHO-EA cells (5000 cells/well) were stimulated with drugs for 4 h at 37 °C in IP1 stimulation buffer containing 0.1% bovine serum albumin. Then, IP1-d2 conjugate and terbium cryptate-labeled anti-IP1 antibody in lysis buffer were added to each well according to manufacturer’s instructions. Plates were incubated at room temperature for > 1 h and then time-resolved fluorescence resonance energy transfer (TR-FRET) signal was measured. The binding of β-arrestin to hOX2R was assessed using a PathHunter assay kit (DiscoveRx, Fremont, CA, USA). Intracellular phosphorylation of ERK1/2 (Thr202/Tyr204) and CREB (Ser133) was measured using a phospho-ERK assay kit (PerkinElmer Inc., Waltham, MA, USA) and a phospho-CREB assay kit (PerkinElmer Inc.), respectively. After 2 h of incubation with each antibody at room temperature, TR-FRET signals were detected. The responses to 0.3% DMSO without or with 1 μM OX-A were used as 0% or 100% responses, respectively. All in vitro data were analyzed using GraphPad Prism (San Diego, CA, USA) or Xlfit (IDBS, Boston, MA, USA), and EC_50_ values were calculated by logistic regression analysis from the data expressed as percentage of control.

### In vitro off-target profiling of TAK-861

Activity of TAK-861 on various enzymes, receptors, and ion channels (102 targets in total) was evaluated at Eurofins Panlabs Discovery Services Taiwan, Limited (Taipei, Taiwan).

### Slice electrophysiology study

Immediately following euthanasia by decapitation, slices of the posterior hypothalamus containing TMN were prepared from C57BL/6J mice and used as described previously^15,16^. The EC_50_ was calculated using EXSUS (EP Croit Co., Ltd, Tokyo, Japan).

### Autoradiography study in mice

For autoradiography analysis, whole brains of mice (32 weeks old) were rapidly dissected, immediately frozen in pre-cooled (− 30 to − 20 °C) 2-methylbutane on dry ice and were stored at − 80 °C until use. OX2R autoradiography analysis was performed at Aptuit (Verona) Srl as described previously^33^. Briefly, 14-µm thick coronal sections were mounted on glass slides. The sections were pre-incubated for 15 min in assay buffer (25 mM HEPES, 1 mM CaCl2, 5 mM MgCl2, pH 7.4) and subsequently incubated for 60 min at room temperature in assay buffer with 3 nM [^3^H]-EMPA. After several washes, the sections were air dried and exposed to BAS-TR2025 Fuji imaging plates (Fuji Photo Film Co., Tokyo, Japan) for 9 days. The level of non-specific binding was defined as the binding in the presence of excess amount of OX2R selective antagonist, JNJ-10397049 (final concentration of 10 µM). Autoradiograms were generated with a Phospho-Imager, Bio-image Analyzer BAS5000 (Fuji Photo Film Co.). Radioactivity of the region of interest was analyzed as photostimulated luminescence (PLS) per mm^2^ using computer assisted microdensitometry (MCID basic, Imaging Research, Canada) and was expressed as relative optical density (ROD) values.

### Surgery, data acquisition, and vigilance state determination in mice and monkeys

Implantation of electrodes and EEG/EMG recordings in mice were performed as described previously^15,16^. Implantation surgery of electrodes was performed under anesthesia of 0.3 mg/kg of medetomidine, 4.0 mg/kg of midazolam, and 5.0 mg/kg of butorphanol in saline. EEG/EMG signals were amplified, filtered (EEG 0.5–250 Hz; EMG 16–250 Hz), digitized at a sampling rate of 200 Hz, and recorded using VitalRecorder (Kissei Comtec Co., Limited, Nagano, Japan). Locomotor activity was measured by an infrared activity sensor (Biotex, Kyoto, Japan). SleepSign (Kissei Comtec Co., Limited) was used to automatically classify wakefulness, NREM sleep, or REM sleep in 4-s epochs, based on EEG/EMG/locomotion data, then the time spent in each stage and number and duration of wakefulness episodes were calculated. Each stage was characterized as follows: (i) wakefulness, low-amplitude EEG with high-voltage EMG activities or locomotion score; (ii) NREM sleep, high-amplitude slow-wave EEG with low-voltage EMG activities; and (iii) REM sleep, theta-dominated EEG with EMG atonia.

Radio-telemetry transmitters (TL10M3-D70-EEE, Data Sciences International Inc., New Brighton, MN, USA) were implanted into monkeys at Hamri Co., Limited, as previously described^19,37^. The two EEG electrodes were stereotaxically positioned at the parietal area (5.0 mm posterior to the bregma and 5 mm lateral to the midline, 15 mm posterior to the bregma and 5 mm lateral to the midline) and secured to the cranium with stainless-steel screws in contact with the dura. Bilateral EMG leads were implanted into the back cervical muscles. After at least a 1-month recovery period, cortical EEG/EMG and locomotor activity were recorded using the telemetry system (Dataquest ART system, Data Sciences International Inc.). Wakefulness, characterized by low-amplitude high-frequency EEG with high-voltage EMG activities or locomotion score, was automatically determined in 20-s epochs using SleepSign software.

### Evaluation of sleep/wakefulness states in mice

This study was conducted with a cross-over design. For the sleep phase experiments, TAK-861 or vehicle (i.e., 0.5% MC in distilled water) was orally administered to OX2R KO mice and their WT littermates, or orexin/ataxin-3 mice and their WT littermates at ZT5 (lights-on at ZT0), and then EEG/EMG and locomotor activity were recorded. For the active phase experiments, TAK-861 or vehicle was orally administered to orexin/ataxin-3 mice at ZT12 or orexin-tTA;TetO DTA mice at ZT14, and then EEG/EMG and locomotor activity were recorded. Similarly, vehicle, modafinil, or clomipramine was orally administered to orexin-tTA;TetO DTA mice at ZT14, and then EEG/EMG and locomotor activity were recorded.

In the repeated administration study, orexin/ataxin-3 mice were grouped into three cohorts: control, acute treatment, and sub-chronic treatment groups. In the control group, vehicle was administered to mice once-daily (ZT12) for 14 days. In the acute treatment group, vehicle was administered to mice at ZT12 for 13 days then TAK-861 was administrated at ZT12 on day 14. In the sub-chronic treatment group, TAK-861 was administered to mice at ZT12 for 14 days. EEG/EMG and locomotor activity were recorded on day 14 for all mice.

### Evaluation of cataplexy-like episodes in mice

Murine cataplexy was scored according to the following four criteria: (1) abrupt episode of EMG atonia lasting ≥ 20 s; (2) behavioral immobility during the episode; (3) predominance of theta activity during the episode; and (4) ≥ 40 s of wakefulness preceding the episode. Immobility of mice was confirmed based on lack of locomotor activity measured by an infrared activity sensor instead of video-based determinations to minimize observer-dependent variations. Using video data, we have previously confirmed that our method can accurately detect cataplexy-like episodes in NT1 model mice^21^, and the anti-cataplectic effects of danavorexton and TAK-994 in NT1 model mice assessed this way translated well to clinical efficacy in individuals with NT1^20,21^. Palatable food, such as chocolate, increases the number of cataplexy episodes during the active phase in orexin KO mice^38,39^. Thus, chocolate was used as a trigger of positive emotions to increase the frequency of cataplexy-like episodes in orexin/ataxin-3 mice. Evaluation of drug efficacy on cataplexy-like episodes was conducted with a cross-over design. TAK-861 or vehicle was orally administered to orexin/ataxin-3 mice at ZT12, then milk chocolate (Hershey’s Kisses Milk Chocolate, Hershey, PA, USA) was placed as a trigger of positive emotions in the cage followed by recording of EEG/EMG and locomotor activity. The effect of TAK-861, modafinil, and clomipramine on spontaneous cataplexy-like episodes in orexin-tTA;TetO DTA mice were determined in the absence of chocolate, to minimize emotional stimuli-induced c-Fos induction in the following test. TAK-861, modafinil, clomipramine, or vehicle was orally administered to orexin-tTA;TetO DTA mice at ZT14, and then EEG/EMG and locomotor activity were recorded.

### Evaluation of sleep/wakefulness states in cynomolgus monkeys

This study was conducted with a two-treatment (drug and vehicle), two-period cross-over design. TAK-861 or vehicle was orally administered to cynomolgus monkeys at ZT12, then EEG/EMG and locomotor activity were recorded.

### Whole-brain c-Fos immunohistochemistry

TAK-861, modafinil, clomipramine, or vehicle were administered to orexin-tTA;TetO DTA mice at ZT14 (2 h after lights-off) because effects of lights-off on c-Fos protein expression have been reported to disappear within 2 h^27^. At 2.5 h after drug administration, mice were sacrificed by transcardial perfusion with heparinized saline (4000 units of heparin per 1000 mL saline), followed by 4% paraformaldehyde perfusion. Brains were dissected and post-fixed. Distribution of c-Fos-positive neurons were imaged at Certerra, Inc., (Cold Spring Harbor, NY, USA) using a whole-brain immunohistochemistry procedure called iDISCO and light-sheet fluorescent microscopy^40,41^. The c-Fos-positive neurons were automatically identified by convolutional neural networks and visualized in 3D. The datasets were warped in 3D by affine and B-spline transformation to an average reference mouse brain generated from 96 8-week-old C57BL/6 mice brains, as described previously^42,43^.

### Pharmacokinetic study in mice

Blood was collected from the tail vein using heparinized tube at 0, 0.25, 0.5, 1, 2, 3, 4, and 8 h after administration of TAK-861 at ZT5 in WT mice and orexin/ataxin-3 mice. In the repeated administration study, blood of orexin/ataxin-3 mice was collected at 0, 0.25, 0.5, 1, 2, 4, 8, and 24 h after administration of TAK-861 at ZT12 on day 14. Plasma was separated from the blood samples by centrifugation. Plasma drug concentrations were quantified with high-performance liquid chromatography-tandem mass spectrometry. The lower limit of quantitation was 3 ng/mL for plasma in mice.

### Statistical analysis for in vivo studies

Statistical analysis was performed using EXSUS (EP Croit Co., Ltd). Pairwise differences between groups were identified using a two-tailed paired *t*-test. In experiments with multiple doses of drugs, the statistical differences were analyzed using a two-tailed Williams test (for parametric data) or a two-tailed Shirley-Williams test (for non-parametric data). In the chronic dosing experiment, statistical significance was determined by Tukey’s multiple comparison test. For the time course of wakefulness in cynomolgus monkeys, data were analyzed with repeated measures analysis of variance followed by a post hoc two-tailed paired *t*-test, with multiplicity adjusted using the Holm method. A *p*-value < 0.05 was considered significant. Statistical comparisons for c-Fos study between groups (vehicle vs. TAK-861, vehicle vs. modafinil, vehicle vs. clomipramine) were conducted based on ROIs, as described previously^42^. Eighty-seven ROIs with low c-Fos expression under 10% quantile of mean counts were removed from a total of 879 ROIs, and the 747 ROIs were selected from within the representative 33 brain categories (frontal pole, cerebral cortex; somatomotor areas; somatosensory areas; gustatory areas; visceral area; auditory areas; visual areas; anterior cingulate area; prelimbic area; infralimbic area; orbital area; agranular insular area; retrosplenial area; posterior parietal association areas; temporal association areas; perirhinal area; ectorhinal area; olfactory areas; hippocampal region; entorhinal area; para-subiculum; post-subiculum; pre-subiculum; subiculum; cortical subplate; striatum; pallidum; thalamus; hypothalamus; midbrain; pons; medulla; and cerebellum) based on Allen Brain Atlas (http://api.brain-map.org/api/v2/structure_graph_download/1.json). Pearson’s correlation coefficients and *p*-values were calculated by c-Fos expression (log-transformed counts after adding one count) between two ROIs in all combinations except for self-correlation across subjects in each group. The *p*-values were adjusted by the Benjamini–Hochberg procedure^44^ with the total number of ROI pairs (278,631 pairs). R (version 4.1.2) was used for statistical analysis and visualization with the packages dplyr (version 1.0.7), data.table (version 1.14.2), tibble (version 3.1.6), readxl (version 1.4.0), ggplot2 (version 3.3.5), hrbrthemes (version 0.8.0), ComplexHeatmap (version 2.10.0), RColorBrewer (version 1.1–2), Hmisc (version 4.7–0), and ggbiplot (version 0.55).

### Ethical approval

The care and use of the animals and the experimental protocols used in this study were approved by the Institutional Animal Care and Use Committee of Takeda Pharmaceutical Company Limited. General procedures for animal care and housing were in accordance with the current Association and Accreditation of Laboratory Animal Care International recommendations. All animal experiments were performed in accordance with ARRIVE guidelines.

## Supplementary Information

Supplementary Information.

## Data Availability

The data that support the findings of this study are available from the corresponding author upon reasonable request.
