# Supplementary material for: TAK-861, a potent, orally available orexin receptor 2-selective agonist, produces wakefulness in monkeys and improves narcolepsy-like phenotypes in mouse models
Source: Sci Rep. 2024 Sep 6;14:20838. doi: 10.1038/s41598-024-70594-1 (PMC11379823; doi:10.1038/s41598-024-70594-1)
Supplement: Supplementary file 1 — Supplementary Information. [file 41598_2024_70594_MOESM1_ESM.docx]

# TAK-861, a potent, orally available orexin receptor 2-selective agonist, produces wakefulness in monkeys and improves narcolepsy-like phenotypes in mouse models

Kayo Mitsukawa, Michiko Terada, Ryuji Yamada, Taku Monjo, Tetsuaki Hiyoshi, Masanori Nakakariya, Yuichi Kajita, Tatsuya Ando, Tatsuki Koike & Haruhide Kimura

**Supplementary Information**


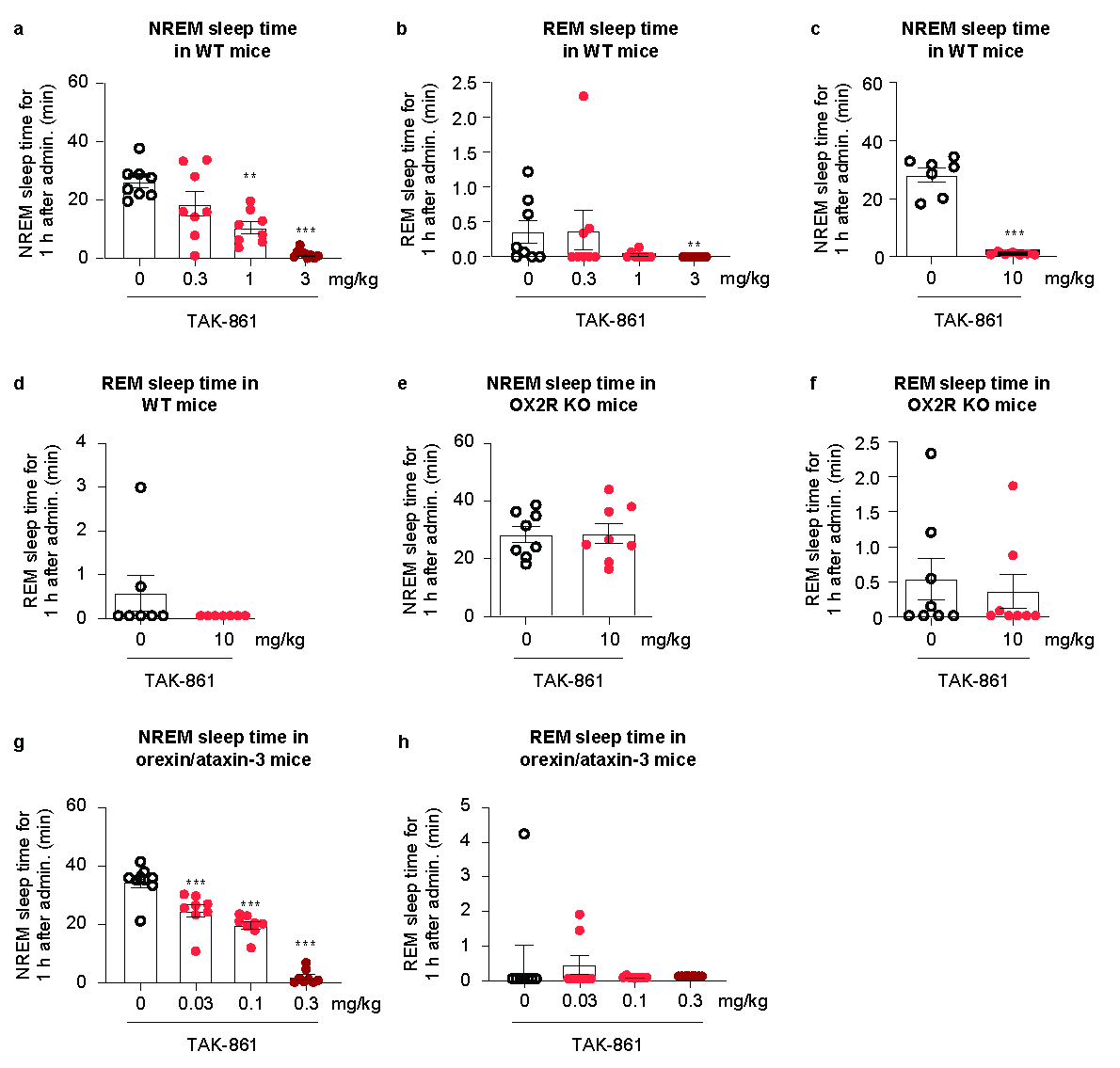


**Supplementary Figure S1.** Effect of TAK-861 on NREM sleep and REM sleep times in WT mice, OX2R KO mice, and orexin/ataxin-3 mice during the sleep phase. Effect of TAK-861 (0.3, 1, and 3 mg/kg, p.o.) or vehicle on (a) NREM sleep time and (b) REM sleep time for 1 h after administration in WT mice. Mean ± SEM; n = 8. ***p* < 0.01, ****p* < 0.001, compared with the vehicle-treated mice, as determined by two-tailed Williams/Shirley-Williams test. Effect of TAK-861 (10 mg/kg, p.o.) or vehicle on NREM sleep time and REM sleep time for 1 h after administration in WT mice (c and d, respectively) and OX2R KO mice (e and f, respectively). Mean ± SEM; n = 7–8. ****p* < 0.001, compared with the vehicle-treated mice, as determined by two-tailed paired *t*-test. Effect of TAK-861 (0.03, 0.1, and 0.3 mg/kg, p.o.) or vehicle on (g) NREM sleep time and (h) REM sleep time for 1 h after administration in orexin/ataxin-3 mice. Mean ± SEM; n = 8. ****p* < 0.001, compared with the vehicle-treated mice, as determined by two-tailed Williams/Shirley-Williams test. KO, knockout; NREM, non-rapid eye movement; p.o., oral administration; REM, rapid eye movement; SEM, standard error of the mean; WT, wild type.


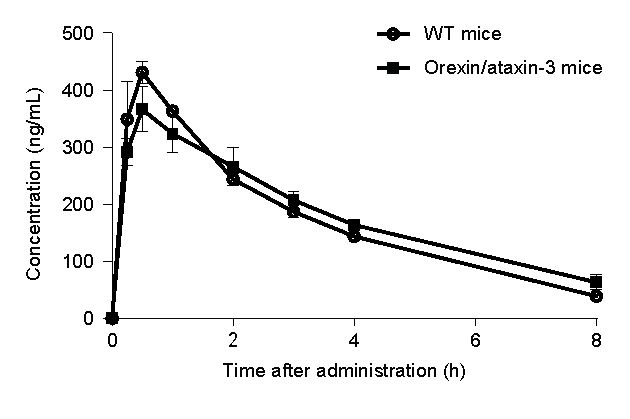


**Supplementary Figure S2.** Time-dependent changes in plasma concentrations of TAK-861 after drug administration in WT mice and orexin/ataxin-3 mice. TAK-861 (1 mg/kg, p.o.) was administered in WT mice and orexin/ataxin-3 mice and blood samples were collected at 0, 0.25, 0.5, 1, 2, 3, 4, and 8 h after administration of TAK-861. Mean ± standard error of the mean; n = 4. p.o., oral administration; WT, wild type.


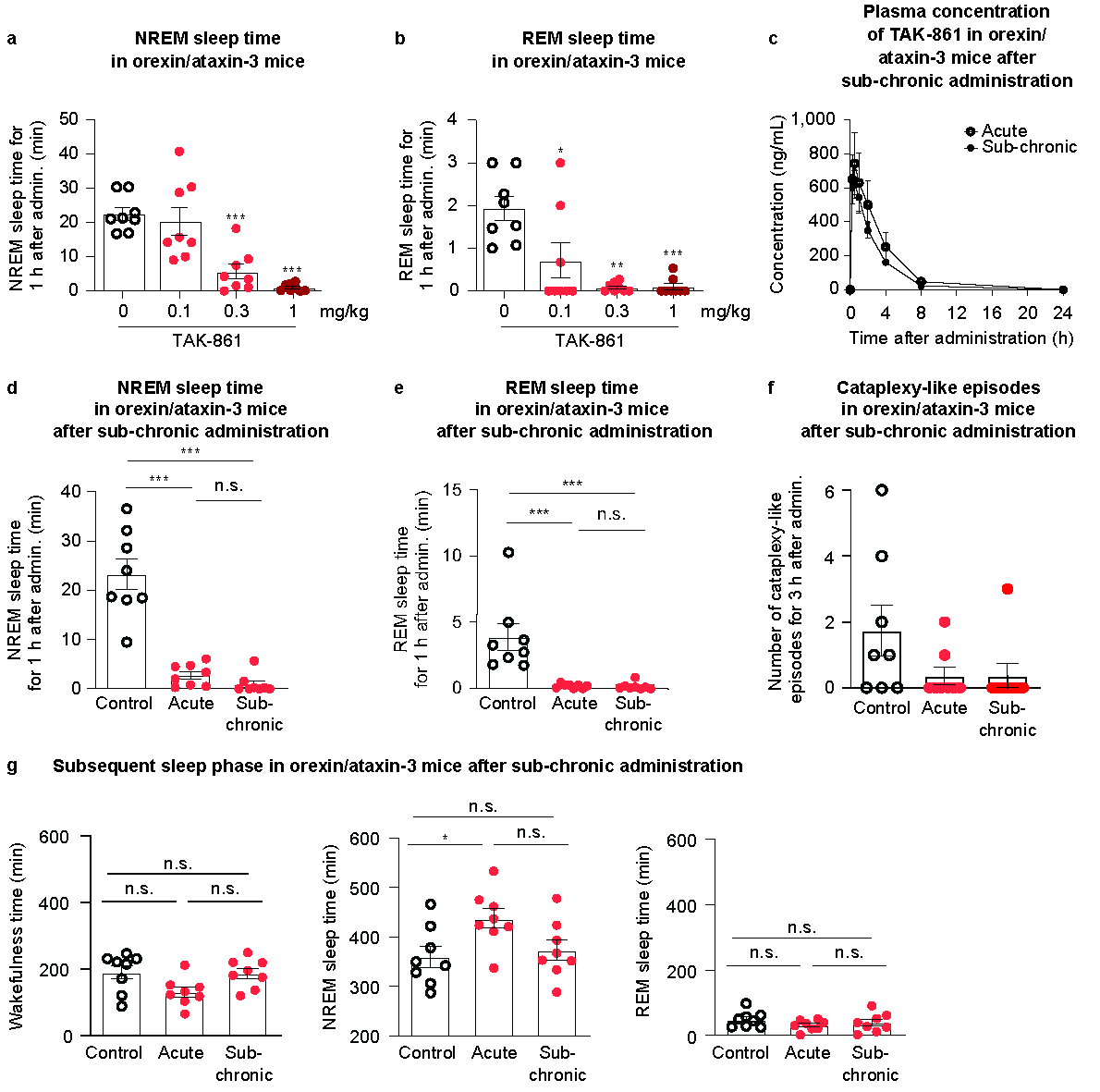


**Supplementary Figure S3.** Effects of TAK-861 on NREM and REM sleep time in orexin/ataxin-3 mice during the active phase. Effect of TAK-861 (0.1, 0.3, and 1 mg/kg, p.o.) or vehicle on (a) NREM sleep time and (b) REM sleep time for 1 h after administration in orexin/ataxin-3 mice. Mean ± SEM; n = 8. **p* < 0.05, ***p* < 0.01, ****p* < 0.001, compared with the vehicle-treated mice, as determined by two-tailed Shirley-Williams test. (c) Time-dependent changes in plasma concentrations of TAK-861 after drug administration in orexin/ataxin-3 mice. TAK-861 (1 mg/kg, p.o.) was administered to orexin/ataxin-3 mice on day 14 after sub-chronic treatment of vehicle or TAK-861 (1 mg/kg, p.o.) for 13 days in acute administration group or sub-chronic administration group, respectively. Blood samples were collected at 0, 0.25, 0.5, 1, 2, 4, 8, and 24 h after administration of TAK-861. Mean ± SEM; n = 4. Effect of TAK-861 (1 mg/kg, p.o., acute or sub-chronic administration) or vehicle on (d) NREM sleep time and (e) REM sleep time for 1 h after administration, on (f) cataplexy-like episodes for 3 h after administration and on (g) wakefulness time, NREM sleep time, and REM sleep time during the subsequent sleep phase (ZT0-10) in orexin/ataxin-3 mice. Mean ± SEM; n = 8. **p* < 0.05, ****p* < 0.001, compared with the vehicle-treated mice, as determined by Tukey’s multiple comparison test. NREM, non-rapid eye movement; n.s., not significant; p.o., oral administration; REM, rapid eye movement; SEM, standard error of the mean.


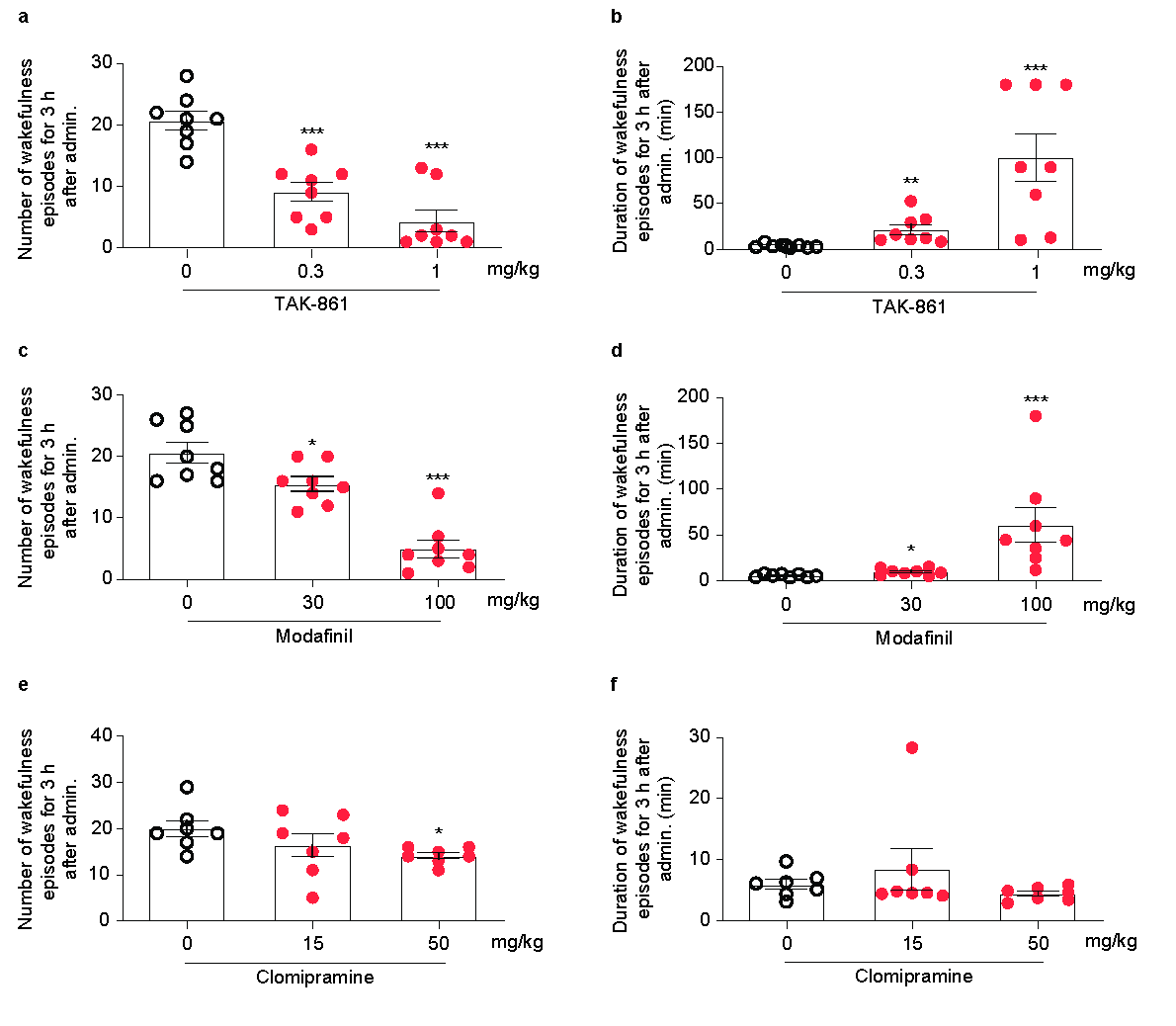


**Supplementary Figure S4.** Effects of TAK-861, modafinil, and clomipramine on wakefulness fragmentation in orexin-tTA;TetO DTA mice during the active phase. Effects of TAK-861 (0.3 and 1 mg/kg, p.o.), modafinil (30 and 100 mg/kg, p.o.), and clomipramine (15 and 50 mg/kg, p.o.) on number of wakefulness episodes (a, c, and e, respectively) and duration of wakefulness episodes (b, d, and f, respectively) in orexin-tTA;TetO DTA mice. Mean ± standard error of the mean; n = 7–8. **p* < 0.05, ***p* < 0.01, ****p* < 0.001, compared with the vehicle-treated mice, as determined by two-tailed Williams/Shirley-Williams test. DTA, diphtheria toxin A.


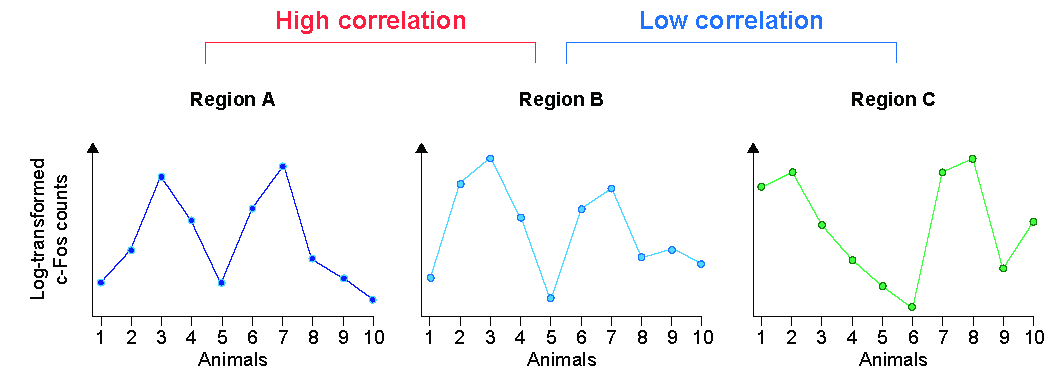


**Supplementary Figure S5.** Schematic drawing of c-Fos expression pattern among mouse brain samples (n = 10) in regions A, B, and C.

**Supplementary Table S1.** Percent inhibition of various enzymes by TAK-861 at 10 μM.

| **Enzymes** | **Percent inhibition** |
| --- | --- |
| 5-Lipoxygenase | –2 |
| ATPase, Ca^2+^, Skeletal Muscle | 16 |
| ATPase, Na^+^/K^+^, Heart | 5 |
| Carbonic Anhydrase II | 1 |
| Catechol-O-Methyl Transferase (COMT) | –2 |
| Cholinesterase, Acetyl | 5 |
| Cyclooxygenase (COX)-1 | 20 |
| Cyclooxygenase (COX)-2 | –6 |
| HMG-CoA Reductase | –3 |
| Monoamine Oxidase (MAO)-A | 3 |
| Monoamine Oxidase (MAO)-B | –9 |
| Nitric Oxide Synthase, Neuronal (nNOS) | 21 |
| Nitric Oxide Synthase, Inducible (iNOS) | 3 |
| Peptidase, Factor Xa | –5 |
| Peptidase, Matrix Metalloproteinase-1 (MMP-1) | –1 |
| Peptidase, Matrix Metalloproteinase-7 (MMP-7) | 0 |
| Peptidase, Matrix Metalloproteinase-13 (MMP-13) | –1 |
| Peptidase, Metalloproteinase, Neutral Endopeptidase | 3 |
| Phosphodiesterase (PDE)10A2 | 1 |
| Phosphodiesterase (PDE)3 | –4 |
| Phosphodiesterase (PDE)4D2 | 7 |
| Phosphodiesterase (PDE)5 | 2 |
| Phosphodiesterase (PDE)6 | –4 |
| Protein Serine/Threonine Kinase, PRKACA (PKA) | –3 |
| Protein Serine/Threonine Kinase, PRKCA (PKCα) | –16 |
| Protein Serine/Threonine Kinase, ROCK1 | 11 |
| Protein Tyrosine Kinase, EGF Receptor | –3 |
| Steroid 5α-Reductase | 3 |
| Xanthine Oxidase | –3 |

**Supplementary Table S2.** Percent inhibition of various receptors or ion channels by TAK-861 at 10 μM.

| **Receptors or ion channels** | **Percent inhibition** |
| --- | --- |
| Adenosine A_1_ | –19 |
| Adenosine A_2A_ | –2 |
| Adenosine A_2B_ | –5 |
| Adrenergic α_1,_ Non-Selective | –4 |
| Adrenergic α_2,_ Non-Selective | –6 |
| Adrenergic β_1_ | –7 |
| Adrenergic β_2_ | 2 |
| Adrenergic β_3_ | 3 |
| Androgen (Testosterone) | –7 |
| Angiotensin AT_1_ | 3 |
| Angiotensin AT_2_ | 15 |
| Bradykinin B_1_ | 4 |
| Bradykinin B_2_ | 13 |
| Calcium Channel L-type, Benzothiazepine | 43 |
| Calcium Channel L-type, Dihydropyridine | 38 |
| Calcium Channel L-type, Phenylalkylamine | 29 |
| Calcium Channel N-type | 2 |
| Cannabinoid CB_1_ | 58 |
| Cannabinoid CB_2_ | 0 |
| Cholecystokinin CCK_1_ (CCK_A_) | 4 |
| Cholecystokinin CCK_2_ (CCK_B_) | 5 |
| Dopamine D_1_ | 13 |
| Dopamine D_2L_ | 13 |
| Dopamine D_2S_ | 14 |
| Dopamine D_3_ | 5 |
| Dopamine D_4.4_ | –11 |
| Endothelin ET_A_ | 21 |
| Estrogen Receptor (Non-Selective) | 9 |
| GABA_A_, Chloride Channel, TBOB | –10 |
| GABA_A_, Flunitrazepam, Central | –25 |
| GABA_A_, Muscimol, Central | –4 |
| Glucocorticoid | 3 |
| Glutamate, AMPA | 24 |
| Glutamate, Kainate | 21 |
| Glutamate, NMDA, Agonism | 14 |
| Glutamate, NMDA, Glycine | –3 |
| Glutamate, NMDA, Phencyclidine | –15 |
| Glycine, Strychnine-Sensitive | 0 |
| Growth Hormone Secretagogue (GHS, Ghrelin) | –2 |
| Histamine H_1_ | 11 |
| Histamine H_2_ | –19 |
| Imidazoline I_2_, Central | –6 |
| Insulin | –9 |
| IP (PGI2) | 3 |
| Melatonin MT_1_ | 11 |
| Muscarinic M_1_ | –9 |
| Muscarinic M_2_ | 0 |
| Muscarinic M_3_ | 0 |
| Nicotinic Acetylcholine α3β4 | –3 |
| Opiate δ_1_ (OP1, DOP) | –2 |
| Opiate κ (OP2, KOP) | 17 |
| Opiate μ (OP3, MOP) | 11 |
| Potassium Channel [K_ATP_] | –10 |
| Potassium Channel [SK_CA_] | 7 |
| Progesterone PR-B | 62 |
| Serotonin (5-Hydroxytryptamine) 5-HT_1A_ | 7 |
| Serotonin (5-Hydroxytryptamine) 5-HT_2A_ | 1 |
| Serotonin (5-Hydroxytryptamine) 5-HT_2B_ | 22 |
| Serotonin (5-Hydroxytryptamine) 5-HT_2C_ | 20 |
| Serotonin (5-Hydroxytryptamine) 5-HT_3_ | –20 |
| Serotonin (5-Hydroxytryptamine) 5-HT_4_ | 9 |
| Sigma, Non-Selective | 20 |
| Sodium Channel, Site 2 | 30 |
| Tachykinin NK_1_ | –14 |
| Tachykinin NK_2_ | –2 |
| Tachykinin NK_3_ | –11 |
| Transporter, Dopamine (DAT) | –1 |
| Transporter, GABA | –1 |
| Transporter, Norepinephrine (NET) | 17 |
| Transporter, Serotonin (5-Hydroxytryptamine) (SERT) | –23 |
| Transporter, Vesicular Monoamine (Non- Selective) | 16 |
| Vasopressin V_1A_ | –18 |
| Vasopressin V_2_ | –18 |

**Supplementary Table S3.** Mean and standard deviation values of the number of c-Fos–positive cells for each group (vehicle, TAK-861, modafinil, and clomipramine) and z-score, *p*-value, and q-value of each comparison (vehicle vs. TAK-861, vehicle vs. modafinil, and vehicle vs. clomipramine) in 747 ROIs. n = 10.
